# Immune status, and not HIV infection or exposure, drives the development of the oral microbiota

**DOI:** 10.1038/s41598-020-67487-4

**Published:** 2020-07-02

**Authors:** M. O. Coker, E. F. Mongodin, S. S. El-Kamary, P. Akhigbe, O. Obuekwe, A. Omoigberale, P. Langenberg, C. Enwonwu, L. Hittle, W. A. Blattner, M. Charurat

**Affiliations:** 1 Department of Oral Biology, Rutgers School of Dental Medicine, 110 Bergen Street, Room C-845, Newark, NJ 07103 USA; 2 Institute of Human Virology, University of Maryland School of Medicine, Baltimore, MD USA; 3 Institute for Genome Sciences, University of Maryland School of Medicine, Baltimore, USA; 4 Department of Epidemiology and Public Health, University of Maryland School of Medicine, Baltimore, MD USA; 5 Institute of Human Virology Nigeria, Abuja, Nigeria; 6 University of Benin Teaching Hospital, Benin, Nigeria; 7 Department of Microbial Pathogenesis, University of Maryland Dental School, Baltimore, MD USA

**Keywords:** Paediatric research, Computational biology and bioinformatics, Microbiology, Molecular medicine

## Abstract

Even with antiretroviral therapy, children born to HIV-infected (HI) mothers are at a higher risk of early-life infections and morbidities including dental disease. The increased risk of dental caries in HI children suggest immune-mediated changes in oral bacterial communities, however, the impact of perinatal HIV exposure on the oral microbiota remains unclear. We hypothesized that the oral microbiota of HI and perinatally HIV-exposed-but-uninfected (HEU) children will significantly differ from HIV-unexposed-and-uninfected (HUU) children. Saliva samples from 286 child-participants in Nigeria, aged ≤ 6 years, were analyzed using 16S rRNA gene sequencing. Perinatal HIV infection was significantly associated with community composition (HI vs. HUU—p = 0.04; HEU vs. HUU—p = 0.11) however, immune status had stronger impacts on bacterial profiles (p < 0.001). We observed age-stratified associations of perinatal HIV exposure on community composition, with HEU children differing from HUU children in early life but HEU children becoming more similar to HUU children with age. Our findings suggest that, regardless of age, HIV infection or exposure, low CD4 levels persistently alter the oral microbiota during this critical developmental period. Data also indicates that, while HIV infection clearly shapes the developing infant oral microbiome, the effect of perinatal exposure (without infection) appears transient.

## Introduction

With the rapid scale up of life-saving antiretroviral therapy (ART)^1^ worldwide, there has been a significant reduction in HIV-related deaths in infants and children^2,3^. Although gaps still remain with respect to infant diagnosis, treatment and follow-up, particularly in resource-limited settings such as sub-Saharan Africa^4^, ART has led to a rising population of infants and children who are either perinatally exposed but uninfected (due to improved prevention of mother to child transmission services) or perinatally infected (due to prolonged survival) with HIV. Children born to HIV-infected mothers—perinatally exposed to HIV—particularly those who eventually acquire the infection, are at risk of acquiring diseases associated with a compromised host immune system, including opportunistic infections^5,6^. In children and adults, HIV infection (and immunosuppression in general) has been associated with increased inflammatory markers^7,8^ and several diseases of the oral cavity, including dental caries^9–15^. Most of these infections are poly-microbial in nature and could be a consequence of immune impairment induced by HIV. The increased risk of developing caries associated with HIV could be attributed to increased colonization of cariogenic bacteria due to immunosuppression, and/or a reduction in salivary flow rate. It has also been suggested that the reduction of CD4^+^ T lymphocytes might lead to the conversion of *Candida* to a pathogenic state, thereby disrupting the oral microbiota^16,17^. With ART, there have been significant and consistent reductions in the prevalence and incidence of oral manifestations of HIV, such as oral candidiasis commonly found in children living with HIV/AIDS^18,19^. However, there is no clear consensus with regards an increased risk of dental caries and other periodontal diseases with HIV. Specifically, we and others^9,11–13^ have observed a higher prevalence of dental caries (particularly in primary dentition) and necrotizing periodontal diseases in children and young adults infected with HIV, while other studies have reported lower caries prevalence and severity compared to the HIV-uninfected individuals^20^. In support of the possible association between HIV infection and oral/dental pathologies, ART has been shown to significantly impact the composition of salivary microbiota^21^; however, there are significant knowledge gaps in describing and explaining the effect of HIV infection or perinatal exposure on salivary microbial composition. This gap further widens with respect to infants and children. Therefore, there is a clear need to examine the relationship between the human oral and salivary microbiota in immunocompromised states like HIV infection and perinatal HIV exposure.

The aim of this study was to compare the salivary microbiota in HIV-infected (HI), HIV exposed-uninfected (HEU) and HIV unexposed/uninfected (HUU) children, using a 16S rRNA gene sequencing strategy. Our group previously reported that compromised immunity is closely associated with caries severity as evidenced by higher decayed-missing-filled primary teeth (dmft) scores among young HI children and children with low CD4^+^ T-cell levels in this study population, highlighting the need to evaluating the microbiota^9^. We therefore hypothesized that perinatal HIV exposure and infection would compromise the immune system and lead to a disruption in the salivary microbiota composition and subsequently causing a shift that could facilitate disease particularly cariogenesis.

## Results

### Participant characteristics

The samples used for this analyses were collected from 286 children (94 HI children, 98 HEU children and 94 HUU children) recruited for cross-sectional comparison. Table 1 provides a summary of the demographic, clinical, maternal and birth characteristics of the participants and how they differed amongst the study groups.

**Table 1:** Characteristics of children whose saliva samples were analyzed and sequenced (N = 286) by study group.

Characteristics	Total (N = 286)		Study group						p value
			HI (N = 94)		HEU (N = 98)		HUU (N = 94)		
	n	(%)	n	(%)	n	(%)	n	(%)	
Age, mean ± SD (range) in months	40.2 ± 21 (8–72)		49.2 ± 17 (13–72)		34.4 ± 18 (8–72)		40.3 ± 21 (8–72)		< 0.0001*
**Age categories**									0.0007*
≤ 36 months	130	(45.5)	29	(30.9)	57	(58.2)	44	(46.8)	
36–72 months	156	(54.5)	65	(69.2)	41	(41.8)	50	(53.2)	
Male	144	(50.3)	53	(56.4)	48	(49.0)	43	(45.7)	0.33
CD4 lymphocyte count, mean ± SD (range) in cells/mm^3^	1,129 ± 554 (0–3,604)		1,021 ± 664 (0–3,604)		1,118 ± 448 (0–2,341)		1,250 ± 512 (0–2,974)		0.02*
**CD4 lymphocyte percent**									0.001*
CD4% < 20	62	(21.7)	27	(28.7)	25	(25.5)	10	(10.6)	
CD4% ≥ 20	224	(78.3)	67	(71.3)	73	(74.5)	84	(89.4)	
On ART treatment	90	(31.5)	90	(95.7)	NA		NA		NA
Presence of any oral pathology, n (%)	65	(22.7)	35	(37.2)	14	(14.3)	16	(17.0)	0.0002*
Caries affected, n (%)	32	(11.2)	17	(18.1)	5	(5.1)	10	(10.6)	0.02*
Antibiotics use in the last 30 days	47	(16.43)	45	(47.9)	1	(1.0)	1	(1.1)	< 0.0001*
**Birth and infant history**									
Induced/artificial membrane rupture	88	(30.8)	21	(22.3)	39	(39.8)	28	(29.8)	0.03*
Caesarean mode of delivery	24	(7.2)	3	(3.2)	14	(14.3)	7	(7.5)	0.02*
Mode of feeding (first 6 months of life)									< 0.0001*
Breast-fed	106	(31.6)	32	(34.0)	34	(34.7)	44	(46.8)	
Formula-fed	65	(19.4)	18	(19.2)	43	(43.9)	21	(22.3)	
Mixed-fed	115	(34.3)	44	(46.8)	21	(21.4)	50	(53.2)	
Duration of breastfeeding, months mean	8.28 ± 6.66 (0–24)		8.85 ± 6.68 (0–24)		3.77 ± 4.81 (0–18)		12.4 ± 5.32 (0–24)		< 0.0001*
Did not complete secondary education	100	(29.9)	43	(45.7)	37	(37.8)	20	(21.3)	0.0003*

p values were derived from Chi and ANOVA/*F* tests (comparison across all groups) were appropriate.

*Statistically significant.

*NA* Not applicable.

By virtue of HIV infection or exposure status, the three groups differed significantly with regards to age, CD4 count and percentage values, oral conditions, duration of feeding and maternal education. Compared to HEU and HUU children, HI children were older, more likely to have lower CD4 lymphocyte counts and percentages, and experienced more dental caries and oral diseases (p < 0.05; Table 1). Regarding birth factors, HI children were also less likely to be delivered after an induced or artificial membrane rupture or caesarean section, and more likely to have been breast fed compared to the other groups (p < 0.05; Table 1). Mothers of HI children were also less likely to complete a secondary education (p < 0.05; Table 1).

Six (6.4%) out of 94 HI children were not receiving ART at the time of enrollment, all of which had a CD4 percent of ≥ 20%.

### Overview of the 16S rRNA gene sequencing dataset

A total of 11,255,328 raw 16S rRNA gene sequencing reads were initially obtained from 290 saliva samples, with an average raw number of sequences per sample being 34,973 (± 11,833 SD, min = 122; max = 78,009). *Cutadapt* was used to trim adapters then DADA2 was implemented to filter, trim, dereplicate, merge paired reads, and remove chimeras (using the built-in *filterAndTrim* function of DADA2 version 1.8 with the following parameters: truncLen = c(235, 200), trimleft = c(3, 0), maxN = 0, maxEE = 5). Post processing, the number of remaining reads for saliva samples ranged from 8,466 to 65,428 per sample. Four samples were subsequently excluded from downstream analyses (three samples had a Good’s estimated coverage < 0.9 and one sample did not have a definite group assignment) and the final sequencing dataset (N = 286 samples) had a total of 11,252,869 sequences (mean 37,234 ± 11,009 SD, min = 1,500; max = 78,009). Trimmed and quality-filtered sequences were finally clustered into 2,434 amplicon sequence variants (ASVs): 2,382 ASVs from 286 saliva samples, and 783 ASVs from 19 plaque samples.

**Figure 1:**
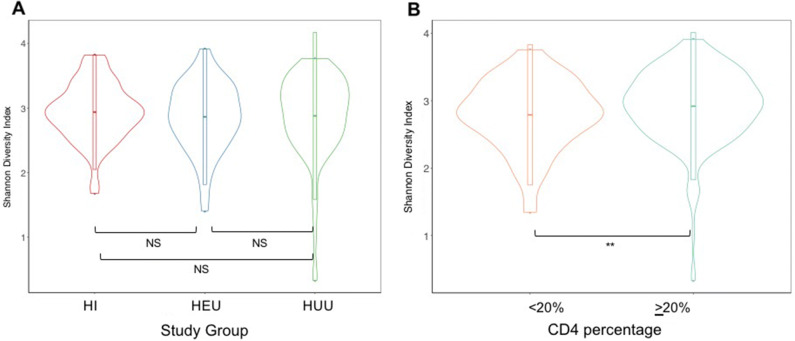
Immune status had a stronger impact on alpha diversity than perinatal HIV exposure/infection. Violin plots showing the distribution of Shannon indices by (**A**). Study group and (**B**). CD4 percent group. No significant differences between study groups were observed while CD4 percentage values were significantly associated with alpha diversity. In the violin plots, the colored dot in the middle of the plot represents the median value, the horizontal bar depicts the interquartile range, and the vertical width of the plot shows the density of the data along the *x*-axis. *p < 0.1, **p < 0.05, ***p < 0.001, *NS* not significant.

Figure 1A shows the distribution of bacterial alpha diversity, as measured using the Shannon diversity index, by study group. In a multivariable regression model (Table S1), Shannon diversity was not associated with any study group (HI, HEU and HUU), but CD4 percent group was an independent factor influencing diversity indices in the entire study population (p = 0.03; Fig. 1B). Further examination of the HI group alone revealed that having CD4 percent value of < 20% (vs. ≥ 20%) remained significantly associated with lower bacterial diversity indices (p = 0.01; data not shown). There was a statistically significant difference between the underlying distributions of all age categories (ANOVA; p < 0.0001)—Figure S1A. Shannon diversity scores steadily increased from ≤ 24 months to 36 months of age and then remained stable thereafter till 72 months with younger age group (children aged ≤ 36 months) presenting with significantly lower diversity indices (p < 0.001) compared to older children (aged over 36 months old). Given this observation, we then examined the differences in Shannon diversity after stratifying the study groups by age (i.e. ≤ 36 months and > 36 months). In this age-stratified analysis, we observed a larger variation in diversity scores in younger age group (≤ 36 month-olds) with Shannon diversity in HI and HEU children being higher than HUU children in the same age group (p = NS; Figure S1B, C).

**Figure 2:**
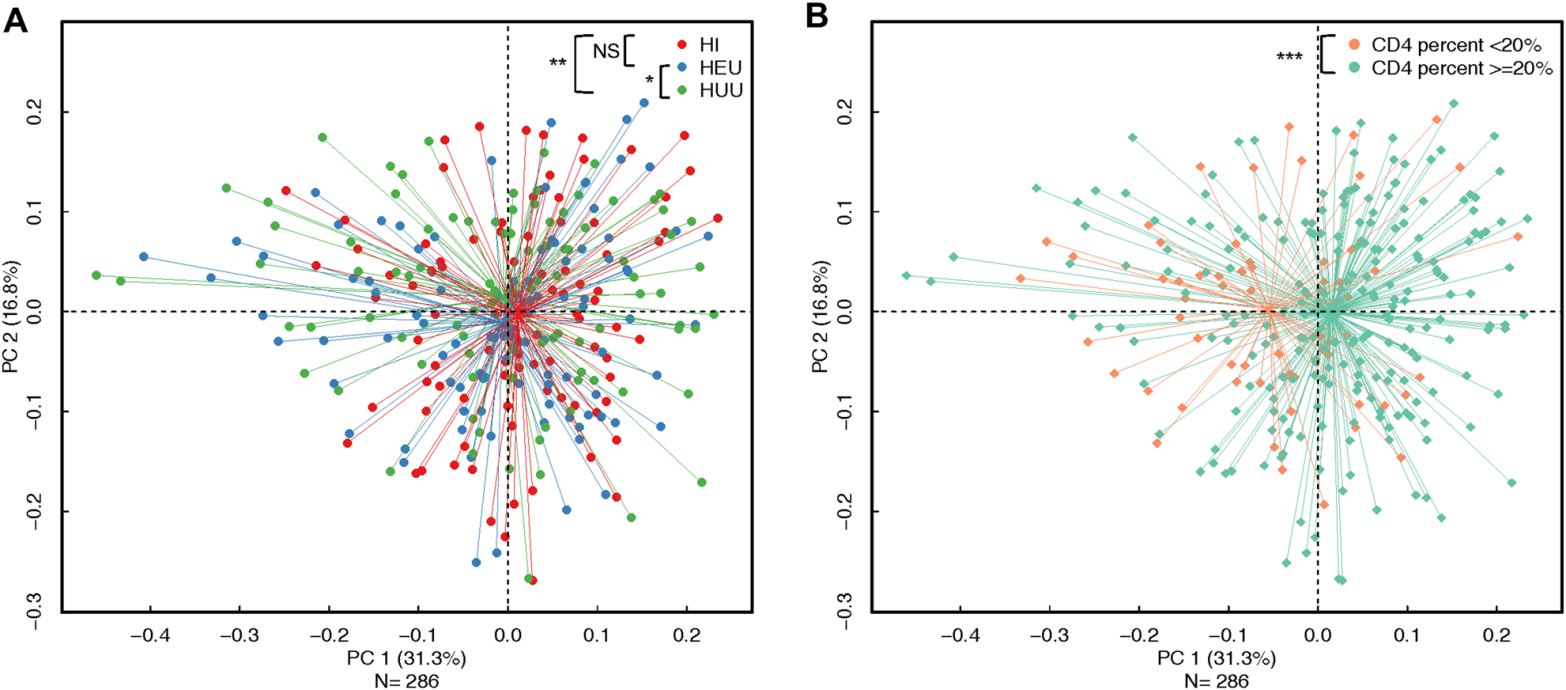
Immune status was more strongly associated with salivary bacterial composition and structure than perinatal HIV infection or exposure. Principal Coordinate Analyses plots based on Generalized Unifrac distances at the ASV level. A. Study group and B. CD4 percent group. *p < 0.1, **p < 0.05, ***p < 0.001, *NS* not significant.

Distance-based (generalized UniFrac distances) analyses showed that samples clustered based on HIV exposure and infection (HI vs. HUU—p = 0.04; HEU vs. HUU—p = 0.11; HI vs HEU—p = 0.34; Fig. 2A), and by immune status (p < 0.001—Fig. 2B). A multivariable PerMANOVA model (R^2^ = 0.07) based on generalized UniFrac distances revealed significant differences between HI vs. HUU children (p = 0.04) but did not reach statistical significance for differences between the HI and HEU groups (p = 0.07), and between the HEU and HUU groups (p = 0.11)—Table S2. In addition to age, covariates such as CD4, delivery mode, duration of breast-feeding were independently associated with microbial community composition (Figure S2A–C) as observed in the PerMANOVA model (All p < 0.1, R^2^ = 0.07; Table S2, Figure S2A–C). As these factors were dissimilar across HIV groups defined by HIV infection or exposure (in Table 1), age, delivery mode and breast-feeding duration were considered confounding variables and included in all multivariable analyses. Neither gender (p = 0.31) nor the presence of one or more carious teeth (p = 0.38; Figure S2D) distinguished community profiles with PCoA or PerMANOVA models.

In an age-stratified analyses, the impact of perinatal HIV exposure/infection varied with age (≤ 36 months and > 36 months) as HEU children had more similar communities when compared to HUU children in the older group (Figure S3A–B), but CD4% remained significantly associated with diversity distance matrices in both age groups—Figure S3C–D. However, we observed key differences in associations with early risk factors with a significant effect of delivery mode and infant feeding in younger children while no statistically significant effect in children aged > 36 months (data not shown). Additionally, despite the lower prevalence of caries in younger children (Table S3) compared to older children, having one or more carious teeth was shown to distinguish salivary microbial communities in children aged ≤ 36 months (p = 0.02) but this comparison did not reach statistical significance in children > 36 months of age.

At the genus level, prevalent genera include *Streptococcus, Actinomyces, Rothia, Leptotrichia, Prevotella, Veillonella, Neisseria, Porphyromonas, Fusobacterium, Corynebacterium,* and *Granulicatella.* In comparing the age groups, for the entire study population, 65 taxa including several gram positive bacteria such as *Streptococcus sanguinis, Streptococcus peroris, Streptococcus lactarius, Abiotrophia defectiva, Actinomyces naeslundii, Corynebacterium durum* and *Leptotrichia hongkongensis* were in significantly lower relative abundance in older children compared to younger children (all with a q < 0.1, Table S4). The older age group also had significantly higher abundances of predominantly gram-negative taxa including *Rothia mucilaginosa, Prevotella melaninogenica, Porphyromonas pasteri, Gemella sanguinis, Fusobacterium periodonticum* and unclassified *Leptotrichia *sp. (q < 0.1, Table S4).

**Figure 3:**
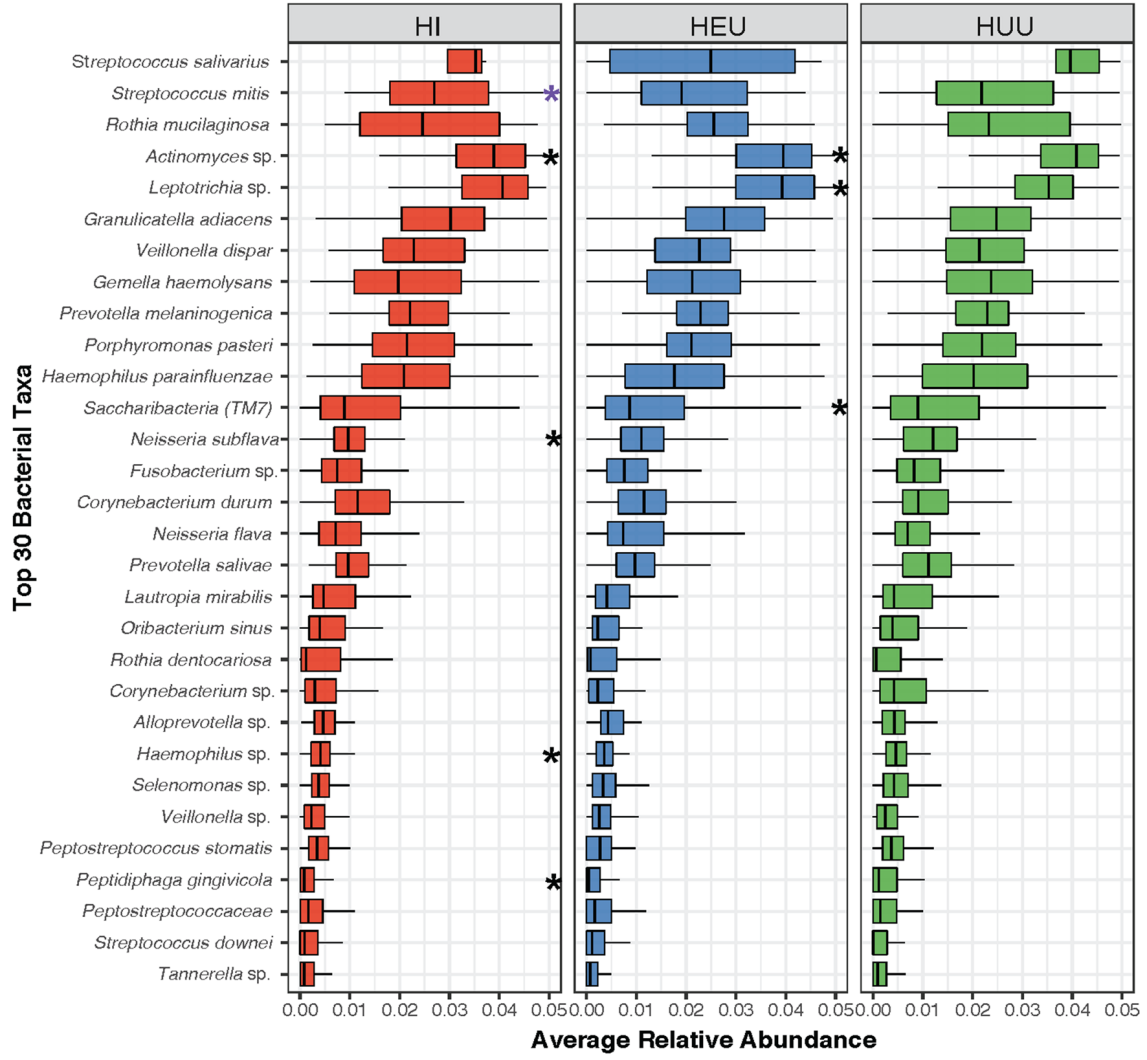
Distribution of Top 30 Bacterial Taxa relative to the three study groups. Individual box plots showing the distribution of relative abundance for each taxa by study group. The black or purple asterisks depict FDR adjusted p (q) values of < 0.1 using MaAsLin2 (black are for comparisons to HUU while purple denotes comparison between HI and HEU). All multivariable models were adjusted for age, delivery mode, breast-feeding duration.

Given these significant differences due to age groups, we then evaluated the effect of HIV infection or exposure using age-adjusted models in subsequent analyses. MaAsLin2 multivariate analyses showed that HI children had significantly lower levels of eight bacterial taxa, including *Actinomyces *and* Neisseria subflava,* while *Corynebacterium diphtheriae* was significantly higher when compared to HUU children (Table 2; Fig. 3)*.* HEU children had lower relative abundance of five taxa, including *Saccharibacteria*, *Selenomonas noxia* and *Actinomyces *sp. while *Streptococcus mutans* and *Leptotrichia *sp. were identified as being significantly higher when compared to HUU children (Table 2). Only three taxa, including *S. mitis* were significantly different when comparing HI to HEU (Table 2; Fig. 3).

**Table 2:** Differentially abundant species due to perinatal HIV infection or exposure. Differentially abundant ASVs in HI and HEU children compared to controls (HI vs. HUU and HEU vs. HUU respectively), and HI vs. HEU children (q < 0.1, using MaAsLin2).

Feature	Metadata	Coefficient	Standard error	p	q	GG taxonomy	HOMD taxonomy
SV1137	HI vs. HUU	− 4.68E−05	1.41E−05	0.001	0.06	uncl. SR1	*Absconditabacteria *(*SR1*) [*G-1*]* bacterium*
SV1150	HI vs. HUU	3.42E−05	1.13E−05	0.003	0.09	uncl. Corynebacterium	*Corynebacterium diphtheriae | HMT-591 | Strain: CIP 100721*
SV238	HI vs. HUU	− 0.000334	0.00011199	0.003	0.09	uncl.[Weeksellaceae]	*Bergeyella *sp.* HMT 322 | Clone: AK152*
SV349	HI vs. HUU	− 0.0001884	6.31E−05	0.003	0.09	uncl. Neisseria	*Neisseria subflava | HMT-476 | Strain: U37*
SV360	HI vs. HUU	− 0.0004741	0.00016087	0.003	0.09	uncl. Haemophilus	*Haemophilus *sp.* HMT 036 | Clone: 16slp93-3e06.p1k*
SV528	HI vs. HUU	− 0.0002929	9.96E−05	0.003	0.09	uncl. Selenomonas	*Selenomonas sputigena | HMT-151 | Strain: ATCC 35185*
SV536	HI vs. HUU	− 0.0001456	5.00E−05	0.004	0.09	uncl. Actinomyces	*Actinomyces *sp. *HMT 180 | Strain: Hal-1083*
SV799	HI vs. HUU	− 9.89E−05	3.02E−05	0.001	0.06	*Selenomonas noxia*	*Selenomonas noxia | HMT-130 | Clone: CI002*
SV91	HI vs. HUU	− 0.0014991	0.00041628	0.0004	0.03	uncl. Actinomycetaceae	*Peptidiphaga gingivicola | HMT-848 | Strain: F0332*
SV462	HI vs. HEU	− 0.0001334	4.29E−05	0.002	0.06	uncl. Streptococcus	*Streptococcus mitis | HMT-677 | Strain: ATCC 49456*
SV526	HI vs. HEU	0.0002244	6.86E−05	0.001	0.05	uncl. SR1	*Absconditabacteria *(*SR1*) [*G-1*]* bacterium HMT-875 | Clone: CN01*
SV565	HI vs. HEU	− 9.95E−05	3.42E−05	0.004	0.09	*Prevotella melaninogenica*	*Prevotella scopos | HMT-885 | Strain: JCM 17725*
SV150	HEU vs. HUU	0.0002472	8.84E−05	0.006	0.08	uncl.[Prevotella]	*Alloprevotella *sp.* HMT 473 | Clone: HF050*
SV345	HEU vs. HUU	− 0.0001509	5.29E−05	0.005	0.07	uncl. Leptotrichia	*NA*
SV536	HEU vs. HUU	− 0.0001337	4.67E−05	0.005	0.07	uncl. Actinomyces	*Actinomyces *sp.* HMT 180 | Strain: Hal-1083*
SV67	HEU vs. HUU	0.0005445	0.00018672	0.004	0.06	uncl. Streptococcus	*Streptococcus mutans | HMT-686 | Strain: NCTC 10449*
SV799	HEU vs. HUU	− 0.0001086	2.6E−05	6.3E−05	0.003	*Selenomonas noxia*	*Selenomonas noxia | HMT-130 | Clone: CI002*
SV80	HEU vs. HUU	− 0.0004727	0.00015103	0.002	0.04	uncl. TM7-3	*Saccharibacteria *(*TM7*) [*G-1*]* bacterium HMT-352 | Clone: DR034*
SV962	HEU vs. HUU	3.70E−05	1.25E−05	0.003	0.06	uncl. Leptotrichia	*Leptotrichia *sp.* HMT 215 Clone: DR011*
SV150	HEU vs. HUU	0.0002472	8.84E−05	0.006	0.08	uncl. [Prevotella]	*Alloprevotella *sp.* HMT 473 | Clone: HF050*

*p* unadjusted p value, *q* FDR-adjusted p value, *GG* Greengenes, *HOMD* Human Oral Microbiome Database, *NA* Not Applicable.

We observed a distinct pattern of correlations between salivary microbial composition and immunological markers. In younger children, higher abundance of *Rothia mucilaginosa* was observed with low CD4 percentage. For children over 36 months of age, having a CD4^+^ T-cell percentage value of < 20% was strongly correlated with a higher abundance of *Actinomyces *sp., *Porphyromonas pasteri* and *Prevotella nanceiensis*.

In order to identify the differentially abundant taxa associated with caries, analyses were carried out in children aged > 36 months only, due to low prevalence of caries in the younger group (Table S3). Presence of caries was positively associated with higher relative abundance of *Actinomyces *sp.,* Rothia mucilaginosa,* and *Capnocytophaga ochracae* (all with a q < 0.1) as well as *Porphyromonas pasteri*, *Prevotella nanceiensis* and *Corynebacterium durum* (p < 0.05 but q > 0.1). Older children who were perinatally exposed to HIV (both HI and HEU children) had higher levels of caries-associated taxa (*Actinomyces *sp. and* Rothia mucilaginosa—*Figure S4).

In the HI group, *Veillonella dispar*, *Actinomyces *sp. and *Selenomonas *sp. were differentially higher in children with caries. Among HI children, untreated HIV-infection (not on ART) was associated with significantly higher levels of *Leptotrichia, Actinomyces, Neisseria* and *Streptococcus salivarius* compared to HI children on ART. Duration on ART did not distinguish salivary microbiota in this cohort. For HEU children, ASVs of *Selenomonas*, *Actinomyces* and *Fusobacterium* were significantly higher in abundance due to caries while *Leptotrichia* was significantly higher in the caries-affected group among HUU children.

## Discussion

To our knowledge, this is the first study to characterize and compare community composition of the salivary microbiota in these three groups (HI, HEU and HUU) of children aged 6–72 months, utilizing next-generation sequencing. Our results suggest that, although there are some taxonomic differences when comparing salivary microbiota of HI and HEU children from unexposed and uninfected counterparts (HUU children), and immunosuppression had a more pronounced effect on salivary bacterial composition. These findings are particularly important given that we performed CD4 assessments on all participants, offering an opportunity to evaluate immunologic markers in these three groups irrespective of HIV infection or exposure status.

Our study is the first of its kind in sub-Saharan Africa and is unique in that blood samples were collected from all participants regardless of their HIV status, to assess the possible association of CD4 on bacterial composition in these groups of children – HI children with relatively well controlled HIV infection, HEU and HUU children. The consistent and significant effect of immunosuppression (CD4 percent values) on the salivary microbiota regardless of age might explain the increased risk for dental caries in HI populations, as we and others have previously reported^9,14,22^. Taxa such as *Actinomyces *sp.,* Rothia mucilaginosa, Porphyromonas pasteri* and *Prevotella nanceiensis* that were moderately or strongly associated with CD4 percent values were also associated with dental caries. Despite the relative uniqueness of our sub-Saharan Africa study population, these observed similarities suggest that our results could be generalizable to other populations infected and/or exposed to HIV.

While some studies^23–29^ have reported significant differences in the bacterial communities comparing lingual and salivary samples in HI with uninfected individuals, others, particularly pediatric studies, have observed no significant differences between HI and HUU children^23,30^. With respect to comparisons between HI and HEU children, Starr et al.^31^ observed fewer health-associated taxa of plaque including *Corynebacterium* in HI youth compared to HEU youth. Recent studies on populations of HEU children^5,32^ have shown an increased risk of early life infections and mortality, as well as higher (although not significant) risk of dental caries^9^ suggesting a less defensive immune system with perinatal HIV exposure. When comparing the three groups, we observed that HEU children, like HI children, had lower CD4 values compared to their unexposed counterparts. Nevertheless, differences in oral microbiota between HEU and HUU children have not been previously reported. By suppressing immune function, perinatal HIV exposure and/or infection could disrupt development of the oral microbiota and drive biological functions related to HIV progression in HI infants and poorer development outcomes in HEU children^33^.

Our study confirms that microbial diversity and community composition significantly differ across age and CD4 percentage values, breast feeding duration and caries groups. These differences confirm previous reports indicating that the oral microbiota changes with age^34^, and that bacterial composition correlates with immune status^35^, breast feeding^36^ and delivery mode^37,38^, although the latter did not reach statistical significance in this study. Importantly, these results also mirror the higher incidence of caries observed in HI children reported by our group and others^9,34^. This further strengthens the hypothesis that complex factors act in concert in shaping the oral microbiota^39^.

The impact of perinatal HIV exposure and infection on the developing oral microbiota varied with age group. With improved prevention-of-mother-to-child interventions in sub-Saharan Africa over the last decade, fewer perinatally exposed children become infected with HIV making for an older population of HI children. Multivariable analytic results (adjusting for age and other confounders) for all participants showed that HI and not HEU children had significantly different communities than HUU children. Within subgroups of age however, HI and HEU appeared to have similar effects in early life while HEU and HUU became more alike at an older age (as shown in Figure S3A, B). This finding supports a recent report by le Roux et al. that suggests that increased risk of morbidities in HEU in early life later could disappear in late infancy particularly in infants who exclusively breast-feed^32^. The association between caries and the salivary microbiota was also age-dependent, as we observed significant associations only in the younger age group (≤ 36 months of age). Similarly, there was some evidence to suggest that the effects of delivery mode, and type and duration of infant feeding (breast-fed vs mixed/formula fed) on the salivary microbiota were more apparent in early life. Overall, as all these early microbial exposures drive immune maturation early in life^40^, it is possible that changes in the developing microbiota will be more apparent in this critical window of development. As regards gender, a similar study of infant oral microbiota^41^ did not detect differences of the oral microbiota when comparing males to females.

The greater abundance of disease-associated taxa such as *Streptococcus mutans, Lactobacillus*, and *Candida* species has been reported in saliva of HIV+ individuals^29,30^. We did not find significant differences in cariogenic *Streptococcus* species including *S. mutans* when comparing caries-affected children to those unaffected. It should be noted that this lack of association has been observed elsewhere^42^, and could be due to the natural history and onset of ECC. Additionally, saliva (compared to supragingival plaque samples) has been shown to be limited in providing high microbial detection levels for cariogenic bacteria. This could explain why, although higher abundances of *S. vestibularis* and *S. mutans* were observed in HI children and in children with caries, these differences were not statistically significant. Furthermore, some species belonging to the *Streptococcus* genus could not be consistently and unequivocally distinguished using 16S rRNA gene sequencing data, therefore could not be taxonomically identified to enable a detectable difference^43,44^. Antibiotic use in the last 30 days before sample collection was highly correlated with HIV infection as HI children were more likely to be prescribed cotrimoxazole as prophylaxis against opportunistic infections (Table 1). This has a potential to mask any significant differences. Furthermore, several authors have found that there are other species that are associated with caries^45,46^. Kistler et al. reported a statistically significantly lower proportion of *Streptococcus mitis* but a higher proportion of *Haemophilus parahaemolyticus* in HIV+ individuals on HAART^23^. Although ART has been implicated as driving these observed differences in the oral microbiome^26,47^, there is evidence to suggest a significant difference in the oral microbiomes in HI individuals, both before and after HAART, in comparison with HIV-uninfected controls^26,48^.

Our cross-sectional evaluation limits the extent to which we can explain our findings. We were unable to observe and measure clinical and environmental changes or account for intra-person variability over time. Larger longitudinal studies, involving supra—and subgingival plaque collection, and viral load assessments (for HI children) while accounting for temporal changes over time are required. Beyond taxonomic characterization, there need to identify bacterial pathways and resulting metabolites that promote health and disease^49^.

Although we attempted to enroll children who were similar in age, the HEU children were significantly younger than the HI or the HUU group. It was challenging to identify and enroll older HEU patients (similar in age to HI and HUU children) for comparison in this study. Once a child has been diagnosed as uninfected, parents or guardians are less likely to bring them to the center for well-baby visits. This resulted in most of the HEU children attending UBTH, our study site, being younger and in need of specialized care due to their history of perinatal infection or immediate follow up after early infant diagnosis. It is possible that even after adjusting for age in a multivariable analysis, residual confounding by age might further explain differences in abundance at all taxonomic levels.

In conclusion, oral microbes maintain a delicate balance during health but this balance might be compromised with immunosuppression leading to conditions (like dental caries) that could manifest in the oral cavity. There is evidence of dysbiosis of the oral microbiota in immunocompromised children. However, the higher caries prevalence is not completely attributable to this dysbiosis. There are other significant factors that play a role in the development of caries therefore additional longitudinal studies are needed to evaluate temporal microbial, behavioral and environmental changes that could increase the risk of caries and other dental diseases. Our results present differences in the salivary microbial communities based on perinatal HIV infection or exposure in terms of diversity and taxonomy. Although still in its infancy, our understanding of the immune-microbiota relationships is necessary for disease prevention and treatment. Oral microbiomes show a wide variation among individuals even more so in the developing child. Differences in taxonomic community composition do not always correlate with distinct gene expression and functional profiles indicating a clear need for large scale prospective studies with a broader scope (including metagenomics, metatranscriptomics and metabolomics) to elucidate the effect of perinatal HIV exposure, infection, and subsequent treatment on the composition and function of the oral microbiota.

## Methods

### Study population

This cross-sectional study was conducted at the University of Benin Teaching Hospital (UBTH) in Benin City, Nigeria and approved by institutional ethical review committee at UBTH and the institutional review board at the University of Maryland, Baltimore (UMB) with annual renewals. All methods were performed in accordance with the relevant guidelines and regulations of these institutions. Only children whose parents or guardians provided informed consent were included in the study. All children were living within 100 miles from Benin City at the time of enrollment. Benin is the capital of Edo State and is an urban, cultural hub with a population of approximately 760,000, comprising ethnic groups from across the country and the world because of its cosmopolitan tendencies.

### Ascertainment of HIV infection, perinatal exposure and immune status

Three groups of children were enrolled for this study for comparison;—HI, HEU, and HUU children. Mother–child dyads were recruited from specific UBTH clinics as previously described^33^. Briefly, HI and HEU children were recruited from the HIV/AIDS pediatric clinic or based on referral by mothers attending the adult ART clinic at UBTH. HUU children were recruited from the well-baby/child pediatric clinics. To accurately identify groups, HIV infection or exposure was determined based on review of maternal and child medical records as well as a HIV antibody and PCR (quantitative RNA) confirmatory test of the child-participant at time of enrollment. CD4 lymphocyte count and percent values were obtained using flow cytometry for all children. At the time of this study, viral load assessments were not routinely performed on all HI patients so such data was not collected for this study. Caregivers were interviewed using standardized questionnaires for sociodemographic characteristics of the child, feeding and oral hygiene practices. Medical history was obtained from these interviews and confirmed/resolved by chart review. Maternal information, labor and delivery data, CD4 lymphocyte percentage categories based on CDC’s 2014 case definitions for stages of HIV infection^50^, and medication use were documented from medical records.

### Sample collection and processing

Prior to sample collection, subjects were instructed to refrain from tooth-brushing or using mouth wash 24 h prior to attending the clinic and to avoid eating 2 h before sample collection.

We collected saliva samples from all study participants using salivette swabs and stored them in − 80 °C and shipped on dry ice to the University of Maryland School of Medicine (UM-SOM)—Institute for Genome Sciences (IGS) in Baltimore, Maryland. Total genomic DNA was extracted from each sample using a protocol developed at the IGS and as previously described^51,52^. Briefly, samples were thawed on ice, then enzymatically lysed using two enzymatic cocktails (first with lysozyme, mutanolysin, and lysostaphin, and then proteinase K and SDS), after which the microbial cells were lysed using bead beating with silica beads (Lysing Matrix B, MP Biomedicals) with the FastPrep instrument (MBio, Santa Ana, CA, USA). The total genomic DNA was then further extracted and purified using the Zymo Fecal DNA kit (Zymo Research, Irvine, CA, USA). DNA extraction negative controls were processed in parallel to sample extractions in order to ensure no exogenous DNA was introduced during the process.

#### 16S rRNA gene PCR amplification and sequencing

Prior to sequencing, two universal primers, 319F (ACTCCTACGGGAGGCAGCAG) and 806R (GGACTACHVGGGTWTCTAAT), were used for PCR amplification of the V3V4 hypervariable regions of the 16S rRNA gene^53^ in 96-well microtiter plates using procedures previously published^51,52,54^. The 806R primer included a unique sequence tag to barcode the samples, enabling up to 500 specimens (each with a different barcode) to be multiplexed in each sequencing run. 16S rRNA genes were amplified in 96-well microtiter plates, using procedures previously published at SOM-IGS^51,52^. Negative controls without a template were processed for each primer pair. The presence of amplicons was confirmed using gel electrophoresis, after which the SequalPrep Normalization Plate kit (Life Technologies, Thermo Fisher Scientific, Waltham, MA, USA) was used for clean-up and normalization (25 ng of 16S PCR amplicon was pooled for each sample), before pooling and sequencing using the Illumina MiSeq (Ilumina Inc. San Diego, CA, USA) 300 bp-PE at the IGS Genomics Resource Center (GRC) according to the manufacturer’s protocol.

### Characterization of the oral microbiota using 16S rRNA gene sequencing

16S rRNA reads were initially screened for low quality bases and short read lengths^54^. Paired-end read pairs were then assembled using PANDAseq^55^ and the resulting consensus sequences were de-multiplexed (i.e. assigned to their original sample), trimmed of artificial barcodes and primers, and assessed for chimeras using the dada2 R package (v.1.6.0)^56^. Low quality 5′ bases of the forward reads and 3′ bases of the reverse reads were removed following inspection of quality plots. Error estimation was calculated on all samples (pooled) following dereplication. Following chimera removal (consensus method), taxonomy was assigned independently using the Human Oral Microbiome Database (HOMD, v.10.1)^57,58^ as reference. ASVs were aligned using the DECIPHER package 36 and arranged into a maximum likelihood phylogeny (GTR model with optimization of the proportion of invariable sites and the gamma rate parameter) using the phangorn package (v.2.5.3)^59^. The resulting phylogenetic tree was combined with the table of ASV and merged with sample data for loading into the R statistical software package v.3.5 using the phyloseq R package (v.1.24.2)^60^ for visualization and statistical analyses.

### Statistical analyses

All downstream analyses were performed using R v.3.5 within the RStudio framework. To characterize group differences in demographic and clinical characteristics, associations between categorical variables were assessed using Pearson’s Chi-square or Fisher’s exact test when appropriate. For continuous variables, Student’s *t* tests and/or ANOVA tests were performed. Covariates including age, gender, birth and maternal factors were evaluated and those found to be associated with caries and perinatal HIV infection or exposure were examined for confounding and effect modification in stratified analyses.

To characterize the salivary microbiota, we calculated and visualized the alpha (within sample) diversity for each sample using the Shannon index, a composite measure of bacterial species richness (estimate of the total number of bacterial species present in each sample) and evenness (estimate of the differences in relative abundance of the different species making up each sample). Statistical significance of differences in Shannon diversity between groups was assessed using *F* tests, Student *t* tests and linear regression analyses (with a significance threshold of < 0.05). Diversity plots were generated using the ggplot2 package in R. To assess beta (between sample) diversity, salivary microbiota community composition were visualized using principal coordinate analysis (PCoA) plots of generalized UniFrac distances. Using a midpoint rooted phylogenetic tree, the overall microbiota community differences between samples were tested by permutational multivariate analysis of variance (PerMANOVA—adonis function, vegan package in R—https://github.com/vegandevs/vegan/blob/master/R/adonis.R) of pairwise generalized UniFrac distance matrices, with 999 permutations. All models were adjusted for age to control for confounding.

To identify statistically significant associations between microbial phenotypes (relative abundance of ASVs) between HI vs. HUU and HEU vs. HUU and HI vs HEU was performed using Multivariate Association with Linear Model (MaAsLin2; https://github.com/biobakery/Maaslin2)^61^. MaAsLin2 utilizes multivariable linear modelling (allowing different continuous and categorical covariates) to calculate effect estimates and identify differentially abundant taxa. In this study, age, CD4 percentage, delivery mode, breastfeeding and caries were included as potential confounders in each model when testing the association between HIV infection or exposure and microbial abundance. Before running MaAsLin2, we first filtered out low-abundance ASVs that had relative abundances < 0.01% and were present in less than 10 individuals. Taxa with Benjamini–Hochberg FDR p-values (q values)^62^ lower than 0.1 were generally considered differentially abundant for the associations between dependent and independent variables. Differentially abundant taxa identified by MaAsLin2 are highlighted in the results.

### Ethics approval and consent to participate

The studies described in this manuscript were approved by the institutional review boards (IRB) of the UMB-SOM and all participants provided written informed consent (IRB study number: HP-00055887).

## Supplementary information

Supplementary information 1

## Data Availability

The data are available from the Sequence Read Archive with accession number PRJNA575737.
