# Supplementary material for: Immune status, and not HIV infection or exposure, drives the development of the oral microbiota
Source: Sci Rep. 2020 Jul 2;10:10830. doi: 10.1038/s41598-020-67487-4 (PMC7331591; doi:10.1038/s41598-020-67487-4)

Immune Status, and not HIV infection or exposure, drives the development of the Oral Microbiota

M. O. Coker[1,2], E. F. Mongodin[3], S.S. El-Kamary[4], P. Akhigbe[5], O. Obuekwe[6], A. Omoigberale[6], P. Langenberg[4], C.Enwonwu[7], L. Hittle[3], W. Blattner[2,4], M. Charurat[2]

[1]Departments of Oral Biology and Epidemiology, Rutgers School of Dental Medicine; [2]Institute of Human Virology, University of Maryland School of Medicine, Baltimore, MD; [3]Institute for Genome Sciences, University of Maryland School of Medicine, Baltimore; [4]Department of Epidemiology and Public Health, University of Maryland School of Medicine, Baltimore, Maryland; [5]Institute of Human Virology, Abuja, Nigeria; [6]University of Benin Teaching Hospital, Benin, Nigeria; [7]University of Maryland Dental School, Baltimore, Maryland.

Table S1. Model statistics from a multivariable regression of alpha diversity indices
Table S2. Associations between HIV status, immune status and other characteristics on salivary bacterial communities.
Table S3. CD4 percentage and Caries status by Age category.
Table S4. Differentially abundant species due to age. Differentially abundant ASVs comparing >36 months of age to < 36 months of age ($q < 0.1$, using MaAsLin2).

Supplementary Figure 1. Distribution of diversity indices differed by age. Age-stratified alpha diversity plots depicted by Shannon index.
A. All participants by age group **B.** Children $\leq$ 36 years of age, by study group
C. Children > 36 years of age, by study group**.**

Supplementary Figure 2A-D. Community composition was significantly associated with age, delivery mode, and infant feeding method, but not caries status. Principal Coordinate Analyses Plots based on Generalized Unifrac distances at the ASV level. **A.** Age group **B.** Delivery mode **C.** Early feeding groups **D.** Caries. Each sample is represented by a solid circle. Circles in different colors represent the groups being compared. PERMANOVA multivariable model:- HIV groups, age, CD4 percent, delivery mode, antibiotics, caries and duration of breastfeeding * $p<0.1$, ** $p<0.05$, *** $p<0.001$, NS not significant.

Supplementary Figure 3A-D. Within age-groups, HIV and CD4 remained associated with salivary community composition. Principal Coordinate Analyses Plots based on Generalized Unifrac distances to evaluate associations with HIV and CD4 in distinct age groups. All multivariable models included age, delivery mode, feeding, HIV and CD4 percentages. For HIV exposure and infection **A.** $\leq$ 36 months **B.** > 36 months; and CD4 percentage categories **C.** $\leq$ 36 months **D.** > 36 months; NS $p\geq0.1$, * $p<0.1$, ** $p<0.05$, *** $p<0.001$.

Supplementary Figure 4A-C. Children perinatally exposed to HIV (infected and uninfected) shared similar age-associated taxa. Based on MaAsLin2 results, salivary bacterial communities of older children (versus younger) exhibit enrichment of several taxa including species of genera *Rothia* and *Actinomyces* in exposed children (HI and HEU) which were also enriched in carious states. MaAsLin2 model coefficients were plotted for the taxa identified at the ASV level. Significance was set at a false discovery rate $Q < 0.1$ using. All multivariable models were adjusted for age, delivery mode, feeding and CD4 percent values. **A.** HI children **B.** HEU children **C.** HUU children.

Table S1. Model statistics from a multivariable regression of alpha diversity indices

Covariate	Estimate	Std. Error	t value	Pr(>|t|)
HI vs. HUU	0.023	0.0636	-0.362	0.71782
HEU vs. HUU	0.04	0.056	-0.142	0.51782
HI vs. HEU	0.080980	0.057729	1.403	0.16181
CD4 percent (<20% vs. ≥ 20%)	-0.1172	0.048	-2.428	0.01582 *
Age (> 36 vs. ≤ 36 months)	-0.155	0.04	-3.711	0.00025 ***
Gender (Male vs. Female)	-0.04615	0.039563	-1.166	0.24446
Delivery mode (Caesarean vs Vaginal)	0.054650	0.072635	0.752	0.45245
Duration of breast feeding	-0.00474	0.007156	-0.663	0.50819
Caries	-0.1179	0.132740	-0.888	0.37522

Multiple R-squared: 0.1036, Adjusted R-squared: 0.07441
F-statistic: 3.546 on 9 and 276 DF, p-value: 0.0003499

Table S2. Associations between HIV status, immune status and other characteristics on salivary bacterial communities. Model statistics from a multivariable PERMANOVA model.

**Covariate**	**F.Model**	**R2**	**Pr(>F)**
HI vs. HUU	1.3757	0.00469	0.035964*
HEU vs. HUU	1.2425	0.00424	0.111888
HI vs. HEU	1.3456	0.00460	0.075924 .
CD4 percent	2.5617	0.00874	0.000999 ***
Age	5.2887	0.01804	0.000999 ***
Gender	1.0816	0.00369	0.313686
Delivery mode (Caesarean vs Vaginal)	1.3227	0.00451	0.073926 .
>6 months of breast-feeding vs. No breastfeeding	1.4002	0.00478	0.007954 **
≤6 months of breast-feeding vs. No breastfeeding	1.2973	0.00442	0.052917 .
>6 months vs. ≤6 months of breast-feeding*	1.6286	0.00556	0.009990 **
Caries	1.0236	0.00349	0.381618

Table S3. CD4 percentage and Caries status by Age category

	Age Category	
**CD4 percent values**	<=36 months (N=130)	36-72 months (N=156)
<20%	34 (26.1)	28 (17.9)
>=20%	96 (73.9)	128 (82.1)
**Caries prevalence**	2 (0.01)	30 (19.2)

Table S4. Differentially abundant species due to age. Differentially abundant ASVs comparing >36 months of age to < 36 months of age (q < 0.1, using MaAsLin2).

	Feature	Metadata	Coefficient	Standard Error	p value	q value	HOMD taxonomy
**Lower Relative Abundance in Older children**	SV5	>36 vs. ≤36 months	-0.021	0.004	<0.001	<0.001	*Streptococcus sanguinis | HMT-758 | Strain: ATCC 10556 | GB: AF003928*
	SV25	>36 vs. ≤36 months	-0.004	0.001	<0.001	<0.001	*Actinomyces naeslundii | HMT-176 | Strain: NCTC 10301 | GB: X81062*
	SV22	>36 vs. ≤36 months	-0.004	0.001	0.000	<0.001	*Leptotrichia hongkongensis | HMT-213 | Clone: DA069 | GB: AF287811*
	SV13	>36 vs. ≤36 months	-0.003	0.001	0.002	0.018	*Abiotrophia defectiva | HMT-389 | Strain: ATCC 49176 | GB: D50541*
	SV18	>36 vs. ≤36 months	-0.003	0.001	<0.001	0.002	*Actinomyces*
	SV29	>36 vs. ≤36 months	-0.003	0.001	0.003	0.025	*Streptococcus lactarius | HMT-948 | Strain: MV1 | GB: NR_117425*
	SV27	>36 vs. ≤36 months	-0.003	0.001	<0.001	0.001	*Rothia aeria | HMT-188 | Clone: ncd2429e02c1 | GB: JF210891*
	SV17	>36 vs. ≤36 months	-0.003	0.001	0.004	0.034	*Corynebacterium durum | HMT-595 | Strain: F0235a | GB: tbd*
	SV39	>36 vs. ≤36 months	-0.003	0.001	0.001	0.013	*Streptococcus peroris | HMT-728 | Strain: GTC848 | GB: AB008314*
	SV35	>36 vs. ≤36 months	-0.003	0.001	0.001	0.013	*Corynebacterium durum | HMT-595 | Strain: IBS G15036 | GB: Z97069*
	SV24	>36 vs. ≤36 months	-0.002	0.001	0.006	0.045	*Lautropia mirabilis | HMT-022 | Strain: Fredericksen | GB: X73223*
	SV61	>36 vs. ≤36 months	-0.002	<0.001	<0.001	0.003	*Actinomyces*
	SV46	>36 vs. ≤36 months	-0.002	<0.001	0.003	0.031	*Saccharibacteria (TM7) [G-1] bacterium HMT 347 | HMT-347 | Clone: BE109 | GB: AY005446 | Status: Phylotype | Body Site: Oral | Genome: no*
	SV47	>36 vs. ≤36 months	-0.001	<0.001	0.010	0.070	*Streptococcus*
	SV108	>36 vs. ≤36 months	-0.001	<0.001	0.001	0.013	*Lautropia mirabilis | HMT-022 | Strain: Fredericksen | GB: X73223 | Status: Named | Body*
	SV111	>36 vs. ≤36 months	-0.001	<0.001	0.012	0.080	*Actinomyces sp. HMT 180 | HMT-180 | Strain: Hal-1083 | GB: AF385522 | Status: Unnamed*
	SV142	>36 vs. ≤36 months	-0.001	<0.001	0.002	0.019	*Streptococcus intermedius | HMT-644 | Strain: ATCC27335 | GB: AF104671 | Status: Named*
	SV110	>36 vs. ≤36 months	<0.001	<0.001	0.014	0.091	*Lachnoanaerobaculum saburreum | HMT-494 | Clone: IR009 | GB: AY349376 | Status: Named*
	SV263	>36 vs. ≤36 months	<0.001	<0.001	0.002	0.020	*Rothia mucilaginosa | HMT-681 | Strain: DY-18 | GB: NR_074690 | Status: Named | Body Site: Oral |*
	SV296	>36 vs. ≤36 months	<0.001	<0.001	0.008	0.056	*Fusobacterium*
	SV297	>36 vs. ≤36 months	<0.001	<0.001	0.016	0.096	*Haemophilus sputorum | HMT-944 | Strain: CCUG 13788 | GB: JF506642 | Status: Named |*

**Higher Relative Abundance in Older children**	SV238	>36 vs. ≤36 months	<0.001	<0.001	<0.001	0.004	*Bergeyella sp. HMT 322 | HMT-322 | Clone: AK152 | GB: AY008691 | Status: Phylotype |*
	SV247	>36 vs. ≤36 months	<0.001	<0.001	0.017	0.100	*Saccharibacteria (TM7) [G-3] bacterium HMT 351 | HMT-351 | Clone: CW040 | GB: AF385506 | Status: Phylotype | Body Site: Oral | Genome: no*
	SV318	>36 vs. ≤36 months	<0.001	<0.001	<0.001	0.004	*Catonella morbi | HMT-165 | Clone: EZ006 | GB: AF385577*
	SV148	>36 vs. ≤36 months	<0.001	<0.001	0.015	0.091	*Saccharibacteria (TM7) [G-3] bacterium HMT 351 | HMT-351 | Clone: CW040 | GB: AF385506 | Status: Phylotype | Body Site: Oral | Genome: no*
	SV191	>36 vs. ≤36 months	<0.001	<0.001	0.014	0.090	*Ruminococcaceae [G-2] bacterium HMT 085 | HMT-085 | Clone: BU014 | GB: AF385563 |*
	SV200	>36 vs. ≤36 months	<0.001	<0.001	0.006	0.045	*Oribacterium asaccharolyticum | HMT-108 | Strain: F0425 | GB: tbd | Status: Named | Body*
	SV219	>36 vs. ≤36 months	<0.001	<0.001	0.006	0.045	*Gemella sanguinis | HMT-757 | Strain: C24KA | GB: AY005051 | Status: Named | Body Site: Oral | Genome: yes*
	SV198	>36 vs. ≤36 months	<0.001	<0.001	0.006	0.045	*Veillonella dispar*
	SV240	>36 vs. ≤36 months	<0.001	<0.001	<0.001	0.006	*Prevotella sp. HMT 313 | HMT-313 | Clone: FM005 | GB: AF432133 | Status: Unnamed | Body Site: Oral | Genome: no*
	SV134	>36 vs. ≤36 months	<0.001	<0.001	0.014	0.091	*Peptostreptococcaceae [XI][G-1] sulci | HMT-467 | Strain: ATCC 35585 | GB: AJ006963 | Status: Named | Body Site: Oral | Genome: yes*
	SV143	>36 vs. ≤36 months	<0.001	<0.001	0.003	0.025	*Prevotella nanceiensis | HMT-299 | Clone: BI027 | GB: AY005064 | Status: Named | Body Site: Oral | Genome: yes*
	SV230	>36 vs. ≤36 months	<0.001	<0.001	<0.001	0.003	*Prevotella pallens | HMT-714 | Strain: JCM 11140 | GB: NR_113121 | Status: Named | Body Site: Oral | Genome: yes*
	SV119	>36 vs. ≤36 months	<0.001	<0.001	0.004	0.038	*Capnocytophaga gingivalis | HMT-337 | Strain: S3 | GB: AY005073 | Status: Named | Body Site: Oral | Genome: yes*
	SV153	>36 vs. ≤36 months	<0.001	<0.001	0.004	0.034	*Leptotrichia sp. HMT 215 | HMT-215 | Clone: DR011 | GB: AF385518 | Status: Unnamed | Body Site: Oral | Genome: yes*
	SV182	>36 vs. ≤36 months	<0.001	<0.001	0.003	0.025	*Parvimonas*
	SV126	>36 vs. ≤36 months	<0.001	<0.001	0.005	0.043	*Veillonella dispar*
	SV132	>36 vs. ≤36 months	<0.001	<0.001	0.001	0.013	*524_3631 | Veillonella atypica | HMT-524 | Clone: MB5_P17 | GB: DQ003631 | Status: Named | Body Site: Oral | Genome: yes*
	SV158	>36 vs. ≤36 months	0.001	<0.001	<0.001	0.001	*Leptotrichia*
	SV129	>36 vs. ≤36 months	0.001	<0.001	<0.001	0.006	*221FP036 | Leptotrichia sp. HMT 221 | HMT-221 | Clone: FP036 | GB: AF432138 | Status: Unnamed | Body Site: Oral | Genome: no*
	SV167	>36 vs. ≤36 months	0.001	<0.001	<0.001	0.002	*221FP036 | Leptotrichia sp. HMT 221 | HMT-221 | Clone: FP036 | GB: AF432138 | Status: Unnamed | Body Site: Oral | Genome: no*
	SV113	>36 vs. ≤36 months	0.001	<0.001	0.004	0.035	*215DR011 | Leptotrichia sp. HMT 215 | HMT-215 | Clone: DR011 | GB: AF385518 | Status: Unnamed | Body Site: Oral | Genome: yes*
	SV112	>36 vs. ≤36 months	0.001	<0.001	<0.001	<0.001	*681_4690 | Rothia mucilaginosa | HMT-681 | Strain: DY-18 | GB: NR_074690 | Status: Named | Body Site: Oral | Genome: yes*
	SV58	>36 vs. ≤36 months	0.001	<0.001	0.012	0.082	*Solobacterium moorei | HMT-678 | Strain: AHP 13983 | GB: AY044915 | Status: Named | Body Site: Oral | Genome: yes*

**Higher Relative Abundance in Older children**	SV106	>36 vs. ≤36 months	0.001	<0.001	0.000	0.000	*Prevotella melaninogenica | HMT-469 | Strain: ATCC 25845 | GB: AY323525 | Status: Named | Body Site: Oral | Genome: yes*
	SV48	>36 vs. ≤36 months	0.001	<0.001	0.003	0.029	*Actinomyces*
	SV107	>36 vs. ≤36 months	0.001	<0.001	<0.001	0.002	*Lachnospiraceae [G-2] bacterium HMT 096 | HMT-096 | Clone: DO008 | GB: AF385508 | Status: Unnamed | Body Site: Oral | Genome: no*
	SV66	>36 vs. ≤36 months	0.001	<0.001	<0.001	0.004	*Veillonella parvula | HMT-161 | Clone: BU083 | GB: AF366266 | Status: Named | Body Site: Oral | Genome: yes*
	SV53	>36 vs. ≤36 months	0.001	<0.001	0.002	0.025	*Atopobium parvulum | HMT-723 | Strain: ATCC22793 | GB: AF292372 | Status: Named | Body Site: Oral | Genome: yes*
	SV84	>36 vs. ≤36 months	0.001	<0.001	<0.001	0.006	*Stomatobaculum sp. HMT 097 | HMT-097 | Clone: DO016 | GB: AF385510 | Status: Unnamed | Body Site: Oral | Genome: no*
	SV51	>36 vs. ≤36 months	0.001	<0.001	<0.001	0.002	*Fusobacterium periodonticum | HMT-201 | Clone: BS011 | GB: AF432130 | Status: Named | Body Site: Oral | Genome: yes*
	SV90	>36 vs. ≤36 months	0.001	<0.001	<0.001	0.002	*Prevotella melaninogenica | HMT-469 | Strain: ATCC 25845 | GB: AY323525 | Status: Named | Body Site: Oral | Genome: yes*
	SV77	>36 vs. ≤36 months	0.001	<0.001	0.001	0.011	*Prevotella histicola | HMT-298 | Clone: BE073 | GB: AF385551 | Status: Named | Body Site: Oral | Genome: yes*
	SV65	>36 vs. ≤36 months	0.002	<0.001	<0.001	<0.001	*Leptotrichia sp. HMT 417 | HMT-417 | Clone: C3MKM102 | GB: AY278621 | Status: Unnamed | Body Site: Oral | Genome: no*
	SV36	>36 vs. ≤36 months	0.002	<0.001	<0.001	<0.001	*Gemella sanguinis | HMT-757 | Strain: C24KA | GB: AY005051 | Status: Named | Body Site: Oral | Genome: yes*
	SV42	>36 vs. ≤36 months	0.003	<0.001	<0.001	<0.001	*Peptostreptococcus stomatis | HMT-112 | Clone: CK035 | GB: AF287763 | Status: Named | Body Site: Oral | Genome: yes*
	SV26	>36 vs. ≤36 months	0.003	0.001	<0.001	0.001	*Saccharibacteria (TM7) [G-1] bacterium HMT 352 | HMT-352 | Clone: DR034 | GB: AF385520 | Status: Phylotype | Body Site: Oral | Genome: no*
	SV33	>36 vs. ≤36 months	0.003	0.001	0.000	0.001	*Porphyromonas pasteri | HMT-279 | Strain: F0450d | GB: tbd | Status: Named | Body*
	SV8	>36 vs. ≤36 months	0.005	0.001	0.001	0.009	*Gemella haemolysans | HMT-626 | Strain: ATCC 10379 | GB: L14326 | Status: Named*
	SV19	>36 vs. ≤36 months	0.005	0.001	<0.001	<0.001	*Porphyromonas pasteri | HMT-279 | Strain: F0450c | GB: tbd | Status: Named | Body*
	SV21	>36 vs. ≤36 months	0.005	0.002	0.013	0.090	*Rothia mucilaginosa | HMT-681 | Strain: DY-18 | GB: NR_074690 | Status: Named |*
	SV14	>36 vs. ≤36 months	0.006	0.001	<0.001	0.001	*Porphyromonas pasteri | HMT-279 | Clone: CW034 | GB: AY008310 | Status: Named |*
	SV15	>36 vs. ≤36 months	0.006	0.001	<0.001	0.001	*Prevotella melaninogenica | HMT-469 | Strain: ATCC 25845 | GB: AY323525 | Status: Named*
	SV2	>36 vs. ≤36 months	0.021	0.005	<0.001	0.002	*Rothia mucilaginosa | HMT-681 | Strain: DY-18 | GB: NR_074690 | Status: Named |*

Supplementary Figure 1. Alpha diversity plots depicted by Shannon index.
A. All participants by age group **B.** Children ≤ 36 years of age, by study group
C. Children > 36 years of age, by study group**.**

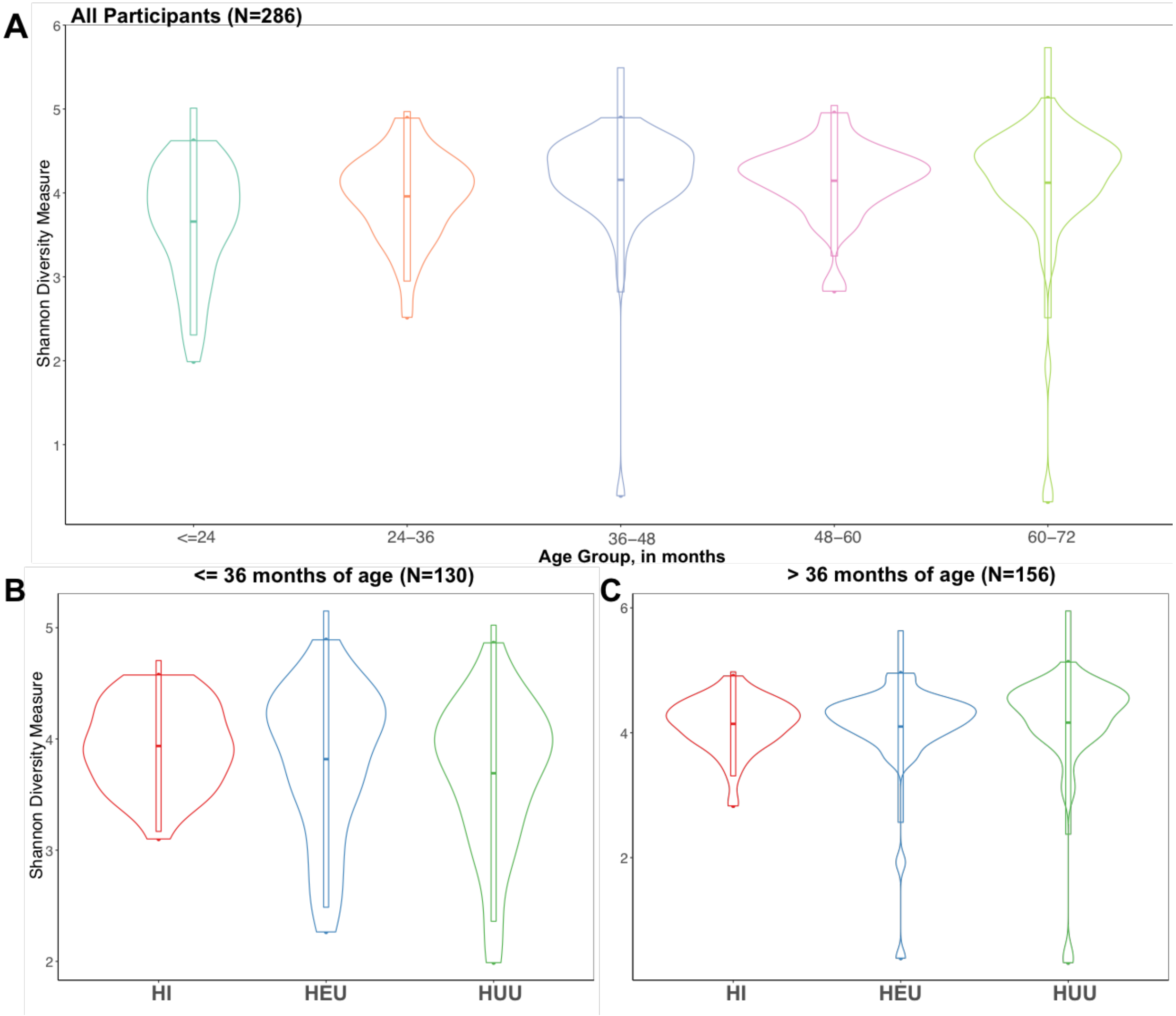

Supplementary Figure 2A-D. Community composition associated with age, delivery mode, infant feeding method and caries status. Principal Coordinate Analyses Plots based on Generalized Unifrac distances at the ASV level. **A.** Age group **B.** Delivery mode **C.** Early feeding groups **D.** Caries. Each sample is represented by a solid circle. Circles in different colors represent the groups being compared. PERMANOVA multivariable model:- HIV groups, age, CD4 percent, delivery mode, antibiotics, caries and duration of breastfeeding * p<0.1, ** p<0.05, *** p <0.001, NS not significant.

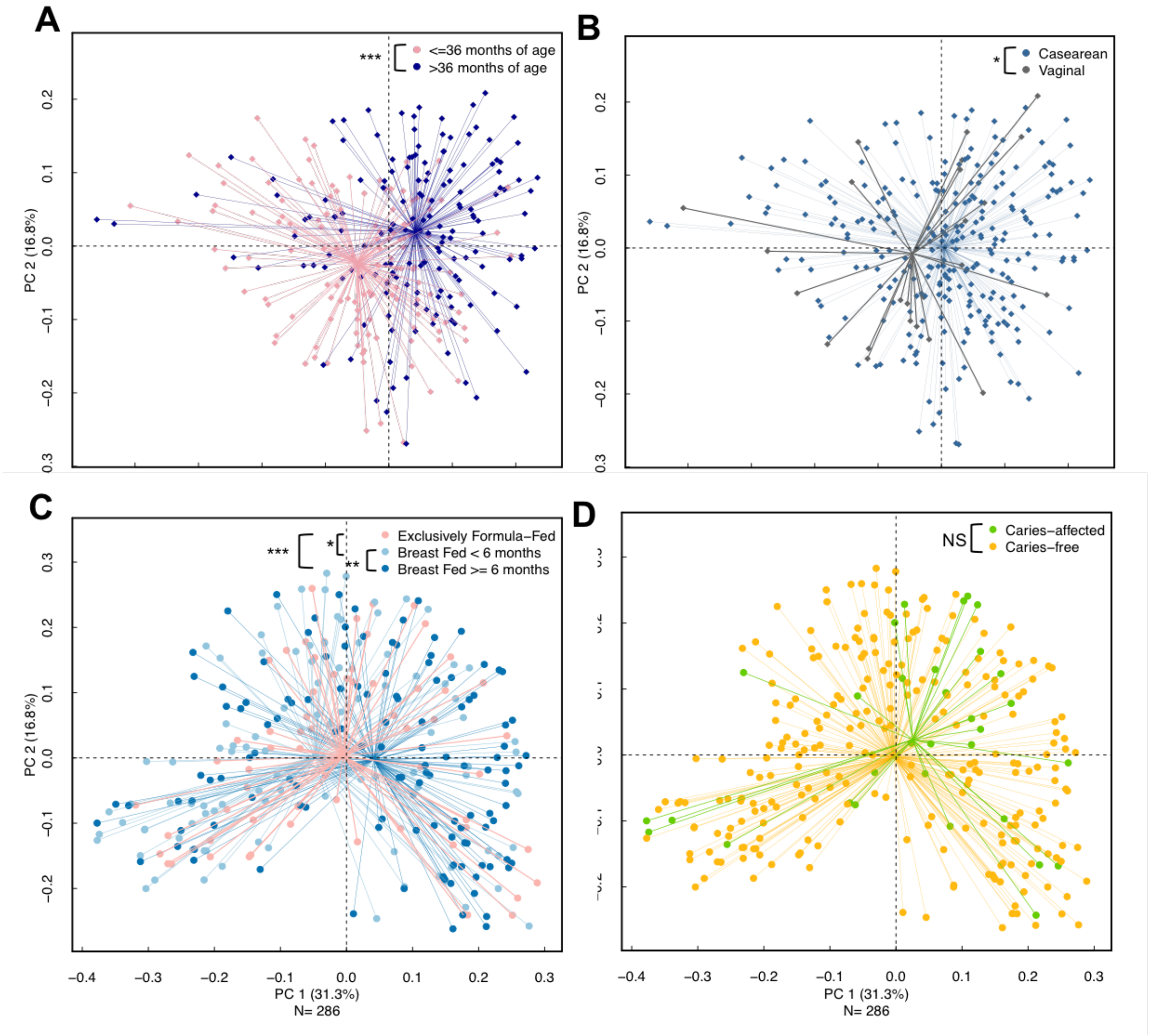

Supplementary Figure 3A-D. Within age-groups, HIV and CD4 remained associated with salivary community composition. Principal Coordinate Analyses Plots based on Generalized Unifrac distances to evaluate associations with HIV and CD4 in distinct age groups. All multivariable models included age, delivery mode, feeding, HIV and CD4 percentages. For HIV exposure and infection **A.** ≤ 36 months **B.** > 36 months; and CD4 percentage categories **C.** ≤ 36 months **D.** > 36 months; NS p≥0.1, * p<0.1, ** p<0.05, *** p <0.001.

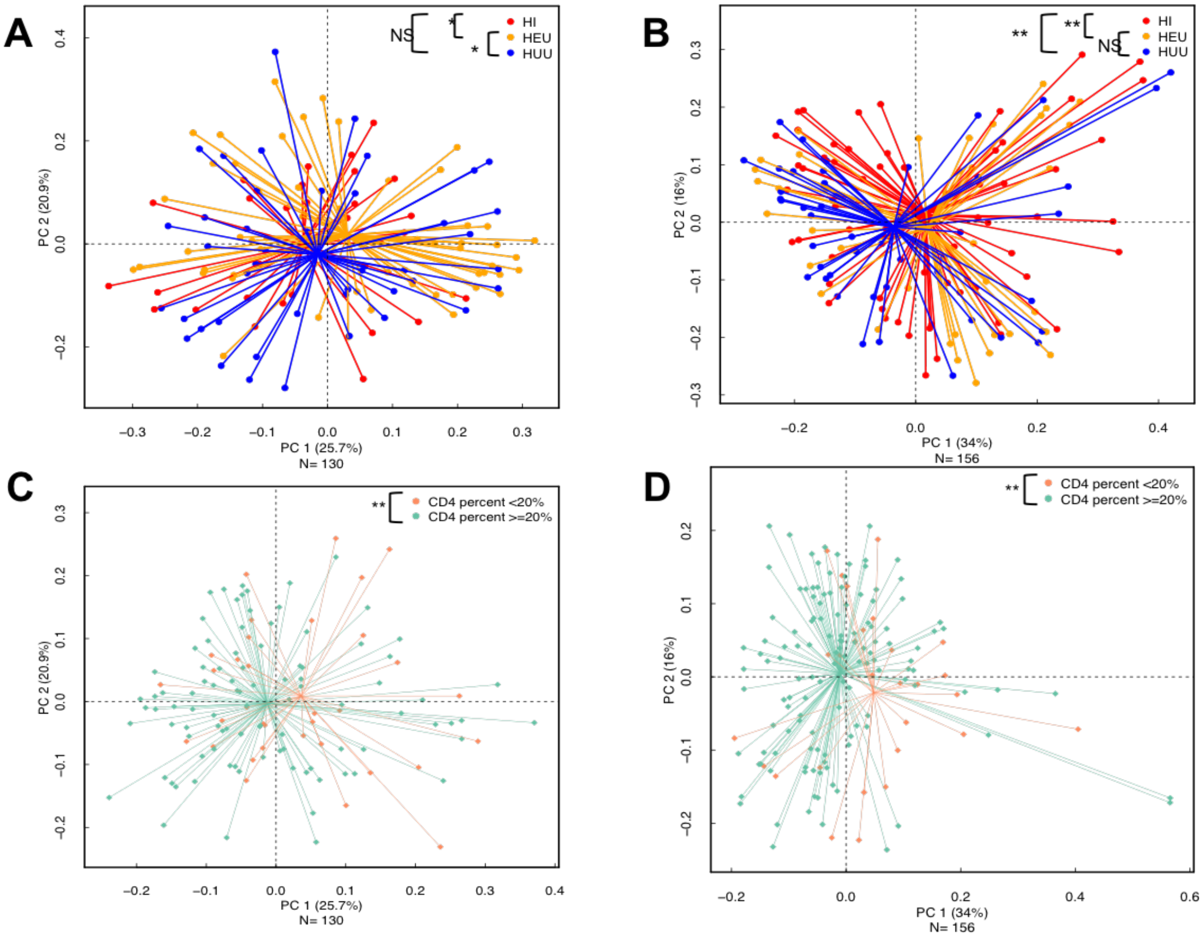

Supplementary Figure 4A-C. Children perinatally exposed to HIV (infected and uninfected) share similar age-associated taxa. Based on MaAsLin2 results, salivary bacterial communities of older children (versus younger) exhibit enrichment of several taxa including species of genera *Rothia* and *Actinomyces* in exposed children (HI and HEU) which were also enriched in carious states. MaAsLin2 model coefficients were plotted for the taxa identified at the ASV level. Significance was set at a false discovery rate $Q < 0.1$ using. All multivariable models were adjusted for age, delivery mode, feeding and CD4 percent values. **A.** HI children **B.** HEU children **C.** HUU children

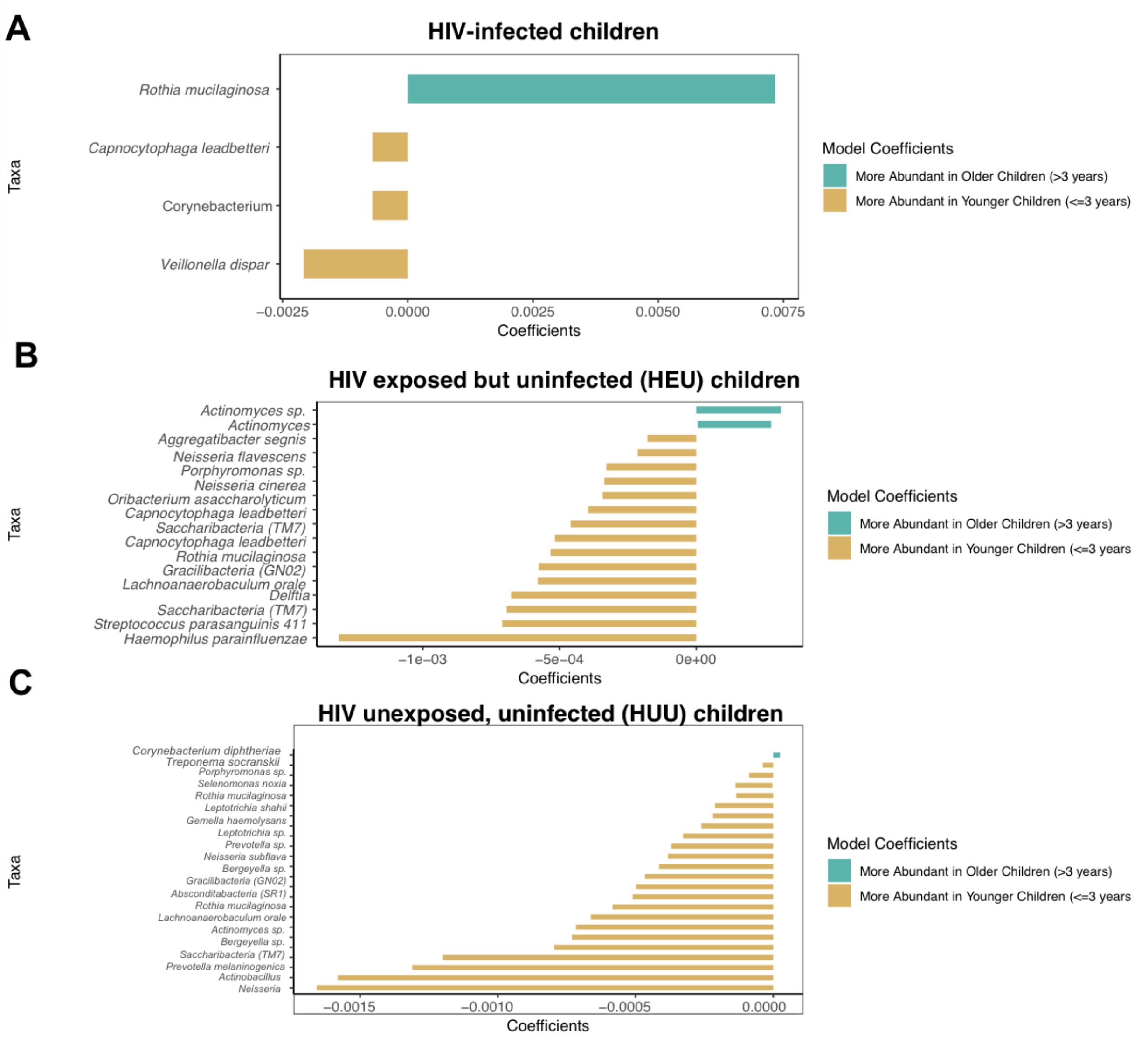

Supplement: Supplementary file 1 — Supplementary information 1 [file 41598_2020_67487_MOESM1_ESM.pdf]
